# Genome-wide meta-analysis of monoclonal gammopathy of undetermined significance (MGUS) identifies risk loci impacting IRF-6

**DOI:** 10.1038/s41408-022-00658-w

**Published:** 2022-04-13

**Authors:** Alyssa Clay-Gilmour, Subhayan Chattopadhyay, Michelle A. T. Hildebrandt, Hauke Thomsen, Niels Weinhold, Pavel Vodicka, Ludmila Vodickova, Per Hoffmann, Markus M. Nöthen, Karl-Heinz Jöckel, Börge Schmidt, Christian Langer, Roman Hajek, Göran Hallmans, Ulrika Pettersson-Kymmer, Claes Ohlsson, Florentin Späth, Richard Houlston, Hartmut Goldschmidt, Elisabet E. Manasanch, Aaron Norman, Shaji Kumar, S. Vincent Rajkumar, Susan Slager, Asta Försti, Celine M. Vachon, Kari Hemminki

**Affiliations:** 1 Arnold School of Public Health, Department of Epidemiology & Biostatistics, University of South Carolina, Columbia, SC USA; 2 Division of Clinical Genetics, Department of Laboratory Medicine, Lund University, Lund, Sweden; 3 Department of Lymphoma/Myeloma, University of Texas MD Anderson Cancer Center, Houston, TX USA; 4 GeneWerk GmbH, Im Neuenheimer Feld 582, 6910 Heidelberg, Germany; 5 Center for Primary Health Care Research, Lund University, Malmo, Sweden; 6 Department of Internal Medicine V, University of Heidelberg, Heidelberg, Germany; 7 Institute of Experimental Medicine, Academy of Sciences of the Czech Republic, Videnska 1083, 142 00 Prague, Czech Republic; 8 Institute of Biology and Medical Genetics, 1st Medical Faculty, Charles University, Albertov 4, 128 00 Prague, Czech Republic; 9 Faculty of Medicine and Biomedical Center in Pilsen, Charles University in Prague, 30605 Pilsen, Czech Republic; 10 Institute of Human Genetics, University of Bonn, Bonn, Germany; 11 Department of Biomedicine, University of Basel, Basel, Switzerland; 12 Institute for Medical Informatics, Biometry and Epidemiology, University Hospital Essen, University of Duisburg-Essen, Duisburg, Germany; 13 Department of Internal Medicine III, University of Ulm, Ulm, Germany; 14 Department of Hematooncology, University Hospital Ostrava and Faculty of Medicine, University of Ostrava, Ostrava, Czech Republic; 15 Department of Public Health and Clinical Medicine, Umea University, Umea, Sweden; 16 Clinical Pharmacology, Department of Integrative Medical Biology, Umea University, Umea, Sweden; 17 Centre for Bone and Arthritis Research, Department of Internal Medicine and Clinical Nutrition, Institute of Medicine, Sahlgrenska Academy, University of Gothenburg, Gothenburg, Sweden; 18 Department of Radiation Sciences, Oncology, Umeå University, Umeå, Sweden; 19 Division of Genetics and Epidemiology, The Institute of Cancer Research, London, UK; 20 National Centre of Tumor Diseases, Heidelberg, Germany; 21 Division of Computational Genomics, Mayo Clinic, Rochester, MN USA; 22 Division of Hematology, Mayo Clinic Rochester Mayo Clinic, Rochester, MN USA; 23 Hopp Children’s Cancer Center (KiTZ), Heidelberg, Germany; 24 Division of Pediatric Neurooncology, German Cancer Research Center (DKFZ), German Cancer Consortium (DKTK), Heidelberg, Germany; 25 Division of Epidemiology, Department of Quantitative Health Sciences, Mayo Clinic, Rochester, MN USA; 26 Biomedical Center, Faculty of Medicine and Biomedical Center in Pilsen, Charles University in Prague, 30605 Pilsen, Czech Republic; 27 Division of Cancer Epidemiology, German Cancer Research Center (DKFZ), Im Neuenheimer Feld 580, D-69120 Heidelberg, Germany

**Keywords:** Genetics, Diseases

**Dear Editor,**

Monoclonal gammopathy of undetermined significance (MGUS) is a benign plasma cell disorder, common in the Western population (3–5% ≥50 years) and characterized by an asymptomatic clonal plasma cell expansion [1]. MGUS progresses to multiple myeloma (MM) at a rate of 1% per year [1], but can also progress to light chain amyloidosis (AL amyloidosis), Waldenström macroglobulinemia, and lymphoma. Familial clustering of MGUS or MM support the role for genetic susceptibility [2]. MM and MGUS have shared heritability, with a genetic correlation of 55% and SNP-based heritability estimates of 17% and 15%, respectively (3,4). This suggests a large portion of missing heritability to be identified. Previous genome-wide association studies (GWAS) have successfully identified 24 common loci associated with MM risk [3, 4]; of these, 12 are also associated with MGUS [5]. Twenty additional loci have been identified for risk of MGUS but the impact of these loci on progression is unknown [5, 6]. Identifying additional common variants contributing to MGUS may elucidate the unaccounted missing heritability for both MGUS and MM. Further, understanding genetic determinants of MGUS are important regardless of MM, given the associations of MGUS with multiple conditions, not just MM. In this study, we performed the largest MGUS GWAS meta-analysis to date and validated associations of known MM/MGUS risk variants.

We included four independent GWAS of MGUS patients and controls (European/European–American) from the United States, Sweden, Germany, and the Czech Republic, described elsewhere [5, 6]. Informed consent was obtained through each study. MGUS cases were primarily identified clinically and defined by the internationally accepted criteria of monoclonal protein concentration <30 g/L, <10% monoclonal plasma cells in the bone marrow, normal plasma calcium, and otherwise asymptomatic features like kidney function, no bone destruction, and no anemia (4,6). However, 33% of MGUS cases from Mayo Clinic were identified via screening alone; sensitivity analysis excluding MGUS identified by screening were performed. Genotyping, imputation, population stratification, principal components analysis, and related quality control steps were performed according to established standards (genotype call rate >90% and imputation quality >0.6) [4, 6]. Common single nucleotide polymorphisms (SNPs) with minor allele frequency (MAF) > 0.01 within each GWAS were analyzed using SNPTEST or PLINK v1.9. Odds ratios (OR) and 95% confidence intervals (CI) were estimated. Summary statistics from all cohorts were meta-analyzed with META using inverse variance weighted random effects linear regression adjusted for imputation call stability. The choice of the statistical design was inspired by observed deviation in MAFs between different studies post quality control. Statistical significance for the GWAS was defined by the conventional threshold, *P* < 5.0 × 10^−8^; SNPs above this value were interrogated further using bionformatic approaches. In addition to GWAS, we performed two analyses including replication of published MM (*N* = 24) and MGUS (*N* = 20) risk loci and the association of identified MGUS variants (at *P* < 1 × 10^−6^) with progressive MGUS, that is progressed to MM. For the latter, we used a case-control design to compare MGUS that was known to progress to MM to MGUS not known to have progressed. Statistical significance was defined as *P* < 0.05 for additional analyses. We meta-analyzed the summary statistics generated from these analyses with METAL. Functional annotation of genetic associations was performed using FUMA (https://fuma.ctglab.nl/), Ensembl Release 104 (May 2021) and HaploReg V4.1 (Broadinstitute.org). In FUMA, expression quantitative trait locus (eQTL) analysis was performed based on cis-eQTLs in blood from 31,684 individuals through the eQTLGen Consortium (https://www.eqtlgen.org/cis-eqtls.html). Further, SNP regions were annotated with the chromatin-state segmentation track (ChromHMM) from Roadmap Epigenome data for all blood, T-cell, hematopoietic stem cells, and B cells. Code for the analyses described herein may be requested from authors.

There was a total of 1738 MGUS cases and 3,755 controls of (European/European–American) included in the meta-analysis (Table S1). After standard GWAS quality control, available SNPs for all platforms ranged from 5.3–6.6 million and were included in the meta-analysis (Table S1).

SNP rs195314 mapping intronic to *CSNK1E* on 22q13.1 was significantly associated with MGUS (OR: 1.35, 95% CI: 1.22–1.49, *P* = 3.66 × 10^−11^) and was consistent in both the European and US cohorts (Figs. 1 and 2, Figs. S1A, B, and Table S2). Sensitivity analysis excluding MGUS identified by screening were performed and revealed a similar association with SNP rs195314 (Meta-analysis: OR = 1.37, 95% CI = 1.16–1.62, *P* = 3.0 × 10^−4^). A limitation of our study is that family history status is unknown for the majority of MGUS cases (EU and MD Anderson). Only 15% of Mayo Clinic MGUS cases had a positive family history of MM/MGUS, and an exploratory analysis of rs195314 restricted to the familial cases and controls, while under-powered, yielded a similar effect and direction of the association (OR = 1.33, 95% CI = 0.90–1.96, *P* = 0.15). rs195314 is in strong linkage disequilibrium (LD) (*r*^2^ > 0.9) with a cluster of SNPs which are cis-eQTLs identified by eQTLgen (Fig. 2). Indicative of function, SNP rs195314 and SNPs in strong LD are located in chromatin states noted as transcriptional sites across several hematological cell types, including primary mononuclear cells, T cells, B cells, and hematopoietic stem cells from peripheral blood (Fig. 2).

**Fig. 1:**
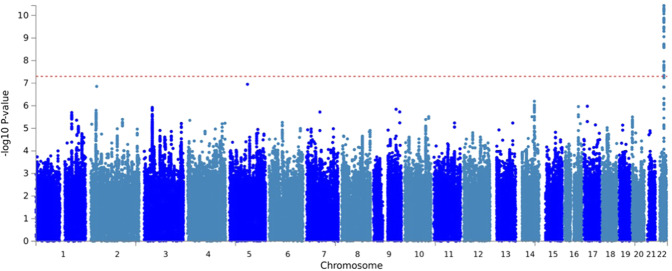
Manhattan plot of the MGUS association. *Y*-axis shows genome-wide P-values (two-sided, calculated using SNPTEST v2.5.2 assuming an additive model) of >6 million successfully imputed autosomal SNPs. The x-axis shows the chromosome number. The red horizontal line represents the genome-wide significance threshold of *P* = 5.0 × 10^−8^.

**Fig. 2:**
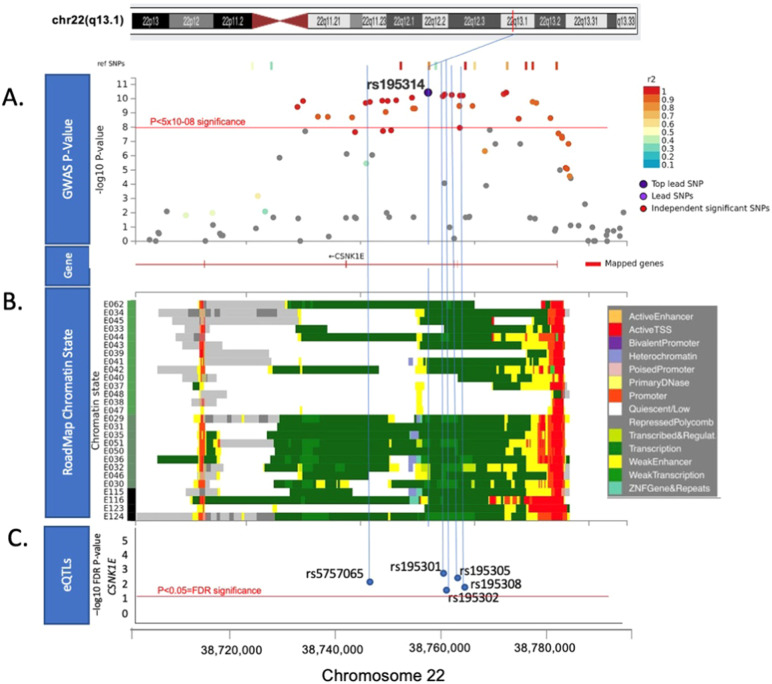
Functional annotation of the rs195314 association. **A** Regional plot of the rs195314 22q13.1 association. **B** Roadmap Epigenome data for all blood, T-cell, HSC, and B cells. E029:Primary monocytes from peripheral blood; E030:Primary neutrophils from peripheral blood; E031:Primary B cells from cord blood; E032:Primary B cells from peripheral blood; E033:Primary T cells from cord blood; E034:Primary T cells from blood; E035:Primary hematopoietic stem cells; E036:Primary hematopoietic stem cells short term culture; E037:Primary T helper memory cells from peripheral blood 2; E038:Primary T help naive cells from peripheral blood; E039:Primary T helper naive cells from peripheral blood; E040:Primary T helper memory cells from peripheral blood 1; E041:Primary T helper cells PMA-Ionomycin stimulated; E042:Primary T helper 17 cells PMA-Ionomycin stimulated; E043:Primary T helper cells from peripheral blood; E044:Primary T regulatory cells from peripheral blood; E045:Primary T cells effector/memory enriched from peripheral blood; E046:Primary Natural Killer cells from peripheral blood; E047:Primary T CD8 naive cells from peripheral blood; E048:Primary T CD8 memory cells from peripheral blood; E-50:Primary hematopoietic stem cells G-CSF mobilized Female; E-51:Primary hematopoietic stem cells G- CSF mobilized Male; E062:Primary Mononuclear Cells from Peripheral Blood; E0116 Lymphoblastic Cell Line. The colors indicate chromatin states imputed by ChromHMM and shown in the key titled “Roadmap Chromatin State”. GWAS significant SNP rs195314 and other strong linkage disequilibrium (LD) SNPs are located in chromatin states noted as transcriptional sites across these hematological cell types. **C** eQTL analysis of *CSNK1E* cis-eQTLs from eQTLgen (https://www.eqtlgen.org/cis-eqtls.html). *Y* axis is -log false discovery rate (FDR) *p* value. *X* axis is base-pair location on chromosome 22. Red line indicates significant FDR *p* value <0.05.

According to Haploreg, rs195314 changes the motif for IRF-6. The core binding site for IRF-6 was defined in the ENCODE analyses as AA(G or C)(T or A)**C**AA which matches our risk allele (the SNP position is bolded) ATGT**C**AA in all but the second nucleotide [7]. Binding strengths of protein-DNA interactions can be measured by position weight matrix (PWM) scores which are calculated for probabilities of any of 4 nucleotides residing in the defined positions [8]. Haploreg cites the ENCODE study (and the related transcription factor database) and gives a PWM score of 11.2 for G allele and of −0.7 for the C allele, implying strong binding of IRF-6 to the reference allele only.

Of the 20 previously identified MGUS risk loci identified by Thomsen et al. [6], we replicated (*P* < 0.05) all but 4 (rs3118053, rs28381958, rs974120, rs10744861) (Table S2). The replication is largely expected given the overlap of cohorts in Thomsen et al. [6] and our study. We also saw associations of MGUS (*P* < 0.05) with 4 of the 24 known MM risk loci rs4487645 (*DNAH11)*, rs6599192 (*ULK4)*, rs7193541 (*RFWD3*), rs34562254 (*TNFRSF13B*) (Table S2). rs195314 is not in linkage disequilibrium (*r*^2^ = 0) with the previously described SNP rs877529 on chr22q13.1 in MM [9]. Given this overlap of genetic variation MM and MGUS, these genes may play an important role in MGUS and MM shared etiology.

In the combined cohorts, there were 165 MGUS cases who progressed to MM and 1,079 known not to have progressed (status unknown for remaining). The average median follow-up time for progression from MGUS to MM was 6.5 years (Mayo Clinic = 5.8 years/Germany = 7.2 years). Unfortunately, we did not have follow-up time for Sweden. The average median age of diagnosis for MGUS patients who eventually progressed was 62, and the median age of progression to MM diagnosis was 67. In our exploratory analyses of 18 SNPs significantly associated with MGUS at *P* < 1.0 × 10^−6^, we found only one MGUS SNP associated with progression to MM (*P* < 0.05). SNP rs12401480 allele C was inversely associated with MGUS progression (OR = 0.90, 95% CI: 0.83–0.98). rs12401480 on chromosome 1 maps to the gene *TDRD5* and impacts motif changes in Mef2 and ZBTB33 (HaploReg), important in histone modification and methylation. According to the Gene Cards database (GeneCards), *TDRD5* is required during spermiogenesis to participate in the repression of transposable elements and prevent their mobilization, which is essential for germline integrity.

None of the other MGUS risk loci identified were associated with progression, including the top MGUS SNP, rs195314 (OR = 1.01, 95% CI: 0.90–1.13, *p* = 0.86). Further, when rs195314 was examined in a GWAS of MM including 4403 MM cases and 7265 controls [10], the OR was 1.02 (95% CI: 0.82–1.24, *p* = 0.5). In the same MM GWAS, the OR for rs12401480 was also null (0.99; 0.97–1.01, *p* = 0.84). Considering the possibility of progression of MGUS to AL amyloidosis, we also interrogated the associations of the MGUS SNPs in a GWAS of 1230 AL amyloidosis patients and 7589 controls, [11] finding no evidence of an association for either rs195314 (OR = 1.01 (0.99–1.02, *p* = 0.87)) or rs12401480 (OR = 0.96 (0.87–1.06, *p* = 0.41)). These data suggest that the two SNPs are not associated with progression of MGUS to MM or AL amyloidosis.

*IRF-6* mutations have been associated with orofacial clefting disorders, including Van der Woude and popliteal pterygium syndromes and genital anomalies, which may be related to disturbances in epidermal differentiation and barrier functions [12]. IRF-6 is expressed in white blood cells and bone marrow but data on possible hematological functions of IRF-6 are limited. However, the IRF family of transcription factors share structural homology and there is a correlation with DNA binding affinity between IRF-6 and IRF-4; the coefficient is 0.6 [7]. IRF-4 has important regulatory function in MM, including allele-specific regulation of the MYC-interacting gene *CDCA7L* [13, 14]. Rs4487645, one of the MM SNPs also found associated with MGUS in our meta-analysis, creates a binding site for IRF-4 at an enhancer site to *CDCA7L* and negatively influences survival in MM patients [13]. IRF-4 has also been shown to be associated with MGUS-associated diseases of Waldenstrom macroglobulinemia/ lymphoplasmacytic lymphoma LPL at 6p25.3 (rs116446171, near IRF-4) [15] and of amyloidosis (AL) at 7p15.3 (rs4487645) [11].

In summary, our MGUS meta-analysis identified a novel locus (rs195314) which may cause a motif change in the binding site of IRF-6, a member of the interferon regulatory transcription factor family. Limited data on MGUS progression suggest that this SNP is not associated with progression to MM or AL amyloidosis. Well-powered studies are needed for validation of these results and to identify further genetic variation contributing to MGUS risk in populations of European and non-European ancestry.

## Supplementary information

Supplemental Material

## Data Availability

Summary statistics are available from the authors.
