# Supplementary material for: Genome-wide meta-analysis of monoclonal gammopathy of undetermined significance (MGUS) identifies risk loci impacting IRF-6
Source: Blood Cancer J. 2022 Apr 13;12(4):60. doi: 10.1038/s41408-022-00658-w (PMC9007981; doi:10.1038/s41408-022-00658-w)

**Supplemental Material**

| **Table S1. Study Characteristics for four studies included in MGUS meta-analysis** | | | | | | | | | | | | | |
| --- | --- | --- | --- | --- | --- | --- | --- | --- | --- | --- | --- | --- | --- |
|  | Germany  (N=1,528) | | Czech  (N=864) | | | Sweden  (N=1,476) | | | USA (Mayo Clinic and MD Anderson)  (N=1,625) | | | Total (meta-analysis)  (N=5,493) | |
|  | Cases | Controls | Cases | Controls | Cases | | Controls | Cases | | Controls | Cases | | Controls |
| Total Count  (N %) | 243  (16) | 1285 (84) | 288  (33) | 576  (67) | 461  (31) | | 1,015 (69) | 746  (46) | | 879  (54) | 1738 (32) | | 3755  (68) |
| Known to Progress to MM (N % cases) | 22 (9) | - | - | - | 74 (16) | | - | 69 (4) | | - | 165 (9) | | - |
|  |  |  |  |  |  | |  |  | |  |  | |  |
| Sex (N%) |  |  |  |  |  | |  |  | |  |  | |  |
| Males | 117 (48) | 638 (50) | 146 (51) | 278 (48) | 212 (46) | | 146 (14) | 432 (58) | | 399 (45) | 907 (52) | | 1461 (39) |
| Females | 126 (52) | 647 (50) | 142 (49) | 298 (52) | 249 (54) | | 869 (86) | 314 (42) | | 480 (55) | 831 (48) | | 2294 (61) |
|  |  |  |  |  |  | |  |  | |  |  | |  |
| Age (median, Standard Deviation or range) | 62  (SD: 11 yrs) | 60  (SD: 8 yrs) | 62  (SD: 12 yrs) | 43  (SD: 11 yrs) | 59 | | 56 | 66  (21-97) | | 61  (28-92) | - | | - |
|  | | | | | | | | | | | | | |
| SNP array platform | Illumina Human OmniExpress-12 v1.0 | | Illumina HumanOmniExpressExome8v1.3 | | | Illumina Human Omni1-Quad BeadChips or OmniExpress-12 v1.0 | | | OncoArray | | |  |  |
| Total common SNPS | 9,914,111 | | 9,940,183 | | | 9,976,561 | | | 10, 217,177 | | |  |  |
| Common SNPs after quality control | 7,045,215 | | 7,075,660 | | | 7,044,116 | | | 7,548,718 | | |  |  |
| SNPs used for full meta-analysis | 6,594,142 | | 6,674,419 | | | 6,688,696 | | | 5,364,845 | | |  |  |

| **Table S2. Genome-wide significant (p<5.0x10-8), suggestive (p<5.0x10-5), and established MM/MGUS associations with MGUS risk** | | | | | | | | | | | | | | | | | | | | | | | | | | | | | |  |
| --- | --- | --- | --- | --- | --- | --- | --- | --- | --- | --- | --- | --- | --- | --- | --- | --- | --- | --- | --- | --- | --- | --- | --- | --- | --- | --- | --- | --- | --- | --- |
|  | | | | | | | | **Meta-Analysis** | | | | | | | | | | | | **US Cohort** | | | | | **European Cohort** | | | | |  |
| **rsID** | **CHR** | | **BP** | **MAF** | | | **Risk** | **Odds ratio (CI)** | | **P_value** | | **beta** | | **se** | **Q** | | **P_het** | | | **Odds ratio (CI)** | | | **P_value** | | **Odds ratio (CI)** | | | **P_value** | |  |
| rs195314 | 22 | | 38771840 | 0.4583 | | | G | 1.35  (1.22 - 1.49) | | 3.66E-11 | | 0.3 | | 0.05 | 15.3 | | 0.002 | | | 1.29  (1.11 - 1.5) | | | 1.12E-03 | | 1.39  (1.03 - 1.88) | | | 3.33E-02 | |  |
| rs11159482 | 14 | | 81490842 | 0.0815 | | | C | 1.58  (1.33 - 1.89) | | 6.33E-07 | | 0.46 | | 0.09 | 2.66 | | 0.448 | | | 1.47  (1.08 - 1.99) | | | 1.38E-02 | | 1.66  (1.3 - 2.12) | | | 3.92E-05 | |  |
| rs12934168 | 16 | | 71436778 | 0.3171 | | | T | 1.26  (1.14 - 1.39) | | 1.09E-06 | | 0.23 | | 0.05 | 8.63 | | 0.035 | | | 1.47  (1.25 - 1.74) | | | 3.16E-06 | | 1.16  (1 - 1.35) | | | 5.59E-02 | |  |
| rs10759357 | 9 | | 112442281 | 0.1731 | | | A | 1.32  (1.18 - 1.49) | | 1.43E-06 | | 0.28 | | 0.06 | 4.07 | | 0.254 | | | 1.19  (0.98 - 1.45) | | | 7.67E-02 | | 1.39  (1.19 - 1.63) | | | 2.99E-05 | |  |
| rs1368892 | 2 | | 27054551 | 0.4557 | | | G | 1.23  (1.14 - 1.33) | | 1.59E-06 | | 0.21 | | 0.04 | 1.35 | | 0.717 | | | 1.24  (1.06 - 1.44) | | | 6.82E-03 | | 1.23  (1.11 - 1.37) | | | 7.38E-05 | |  |
| rs117723545 | 9 | | 130682755 | 0.0586 | | | A | 1.92  (1.46 - 2.52) | | 1.87E-06 | | 0.65 | | 0.14 | 0.2 | | 0.652 | | | 1.82  (1.3 - 2.55) | | | 4.65E-04 | | 2.07  (1.34 - 3.2) | | | 1.09E-03 | |  |
| rs12401480 | 1 | | 179601231 | 0.4851 | | | C | 1.22  (1.13 - 1.32) | | 2.00E-06 | | 0.2 | | 0.04 | 6.69 | | 0.083 | | | 1.22  (1.06 - 1.42) | | | 7.06E-03 | | 1.2  (0.99 - 1.45) | | | 6.44E-02 | |  |
| rs73581924 | 20 | | 2717289 | 0.1004 | | | T | 1.4  (1.22 - 1.61) | | 3.13E-06 | | 0.34 | | 0.07 | 1 | | 0.800 | | | 1.49  (1.17 - 1.91) | | | 1.45E-03 | | 1.37  (1.15 - 1.64) | | | 5.59E-04 | |  |
| rs34708946 | 1 | | 203964395 | 0.0586 | | | A | 1.51  (1.26 - 1.8) | | 4.40E-06 | | 0.41 | | 0.09 | 4.29 | | 0.232 | | | 1.66  (1.18 - 2.32) | | | 3.17E-03 | | 1.46  (1.08 - 1.96) | | | 1.34E-02 | |  |
| rs2046396 | 6 | | 66727242 | 0.4165 | | | T | 1.23  (1.12 - 1.36) | | 5.53E-06 | | 0.21 | | 0.05 | 0.55 | | 0.907 | | | 1.18  (1.01 - 1.39) | | | 4.09E-02 | | 1.26  (1.13 - 1.4) | | | 4.05E-05 | |  |
| rs55743322 | 11 | | 103558935 | 0.1779 | | | G | 1.31  (1.16 - 1.47) | | 5.84E-06 | | 0.27 | | 0.06 | 9.56 | | 0.023 | | | 1.59  (1.3 - 1.94) | | | 6.58E-06 | | 1.17  (0.94 - 1.46) | | | 1.64E-01 | |  |
| rs1289390 | 13 | | 99341964 | 0.3718 | | | C | 1.22  (1.13 - 1.32) | | 5.86E-06 | | 0.2 | | 0.04 | 0.82 | | 0.845 | | | 1.26  (1.08 - 1.47) | | | 3.75E-03 | | 1.21  (1.09 - 1.35) | | | 4.58E-04 | |  |
| rs13143485 | 4 | | 187870988 | 0.4171 | | | A | 1.22  (1.13 - 1.32) | | 6.00E-06 | | 0.2 | | 0.04 | 2.75 | | 0.431 | | | 1.14  (0.98 - 1.32) | | | 9.50E-02 | | 1.26  (1.14 - 1.4) | | | 1.38E-05 | |  |
| rs34547986 | 4 | | 173780405 | 0.4185 | | | A | 1.22  (1.11 - 1.35) | | 6.35E-06 | | 0.2 | | 0.05 | 2.24 | | 0.525 | | | 1.27  (1.09 - 1.49) | | | 2.19E-03 | | 1.2  (1.08 - 1.34) | | | 7.61E-04 | |  |
| rs75876447 | 2 | | 5415937 | 0.0974 | | | C | 1.52  (1.28 - 1.82) | | 6.62E-06 | | 0.42 | | 0.09 | 5.17 | | 0.160 | | | 1.25  (0.93 - 1.68) | | | 1.43E-01 | | 1.71  (1.32 - 2.21) | | | 5.13E-05 | |  |
| rs139820445 | 19 | | 19071516 | 0.0815 | | | C | 1.45  (1.24 - 1.69) | | 7.27E-06 | | 0.37 | | 0.08 | 2.61 | | 0.455 | | | 1.32  (1 - 1.73) | | | 4.61E-02 | | 1.6  (1.22 - 2.1) | | | 6.05E-04 | |  |
| rs28872849 | 1 | | 212918924 | 0.1809 | | | T | 1.28  (1.14 - 1.44) | | 8.21E-06 | | 0.25 | | 0.06 | 0.49 | | 0.922 | | | 1.29  (1.06 - 1.57) | | | 1.13E-02 | | 1.28  (1.12 - 1.46) | | | 2.43E-04 | |  |
| rs2440186 | 18 | | 26518728 | 0.1272 | | | T | 1.34  (1.17 - 1.53) | | 9.50E-06 | | 0.29 | | 0.07 | 5.1 | | 0.165 | | | 1.17  (0.91 - 1.48) | | | 2.16E-01 | | 1.44  (1.17 - 1.78) | | | 6.46E-04 | |  |
| **Known MGUS & MM Risk Loci and Association in MGUS GWAS** | | | | | | | | | | | | | | | | | | | | | | | | | | | | | | |
|  | | | | | | | | | | | | | **Meta-analysis** | | | | | | **US Cohort** | | **European Cohort** | | | | | | | | | |
| **Study** | | **rsID** | | | **CHR** | **BP** | | | **Risk Allele** | | **Published**  **P_value** | | **P_value** | | | **beta** | | **se** | **P_value** | | | **beta** | | **se** | | **P_value** | **beta** | | **se** | |
| Thomsen et al. 2019 | | rs3009934 | | | 1 | 214301323 | | | T | | 2.00E-06 | | 9.26E-05 | | | 0.18 | | 0.05 | 9.87E-01 | | | 0.00 | | 0.08 | | 4.17E-03 | 0.29 | | 0.10 | |
| Thomsen et al. 2019 | | rs9848754 | | | 3 | 41753647 | | | T | | 5.60E-07 | | 8.37E-03 | | | 0.16 | | 0.06 | 1.17E-02 | | | 0.26 | | 0.10 | | 2.05E-05 | 0.37 | | 0.09 | |
| Thomsen et al. 2019 | | rs73180532 | | | 3 | 104051156 | | | C | | 6.80E-06 | | 1.66E-05 | | | 0.19 | | 0.04 | 8.70E-02 | | | 0.13 | | 0.08 | | 4.87E-05 | 0.22 | | 0.05 | |
| Thomsen et al. 2019 | | rs72888948 | | | 4 | 108802381 | | | T | | 5.10E-06 | | 5.93E-05 | | | 0.28 | | 0.07 | 6.27E-01 | | | 0.06 | | 0.13 | | 7.33E-06 | 0.38 | | 0.08 | |
| Thomsen et al. 2019 | | rs9656789 | | | 8 | 105068489 | | | A | | 3.40E-06 | | 3.10E-05 | | | 0.23 | | 0.05 | 3.79E-01 | | | 0.09 | | 0.10 | | 9.51E-06 | 0.29 | | 0.07 | |
| Thomsen et al. 2019 | | rs4928692 | | | 8 | 143466597 | | | G | | 2.50E-06 | | 5.50E-03 | | | 0.15 | | 0.06 | 8.01E-02 | | | 0.17 | | 0.10 | | 2.71E-05 | 0.31 | | 0.07 | |
| Thomsen et al. 2019 | | rs7920332 | | | 10 | 7250346 | | | C | | 7.10E-06 | | 6.32E-04 | | | 0.15 | | 0.04 | 6.66E-01 | | | 0.03 | | 0.08 | | 2.53E-04 | 0.24 | | 0.07 | |
| Thomsen et al. 2019 | | rs12436964 | | | 14 | 69108086 | | | T | | 2.40E-06 | | 2.70E-04 | | | 0.17 | | 0.05 | 7.97E-01 | | | 0.02 | | 0.08 | | 7.84E-05 | 0.26 | | 0.07 | |
| Thomsen et al. 2019 | | rs4561409 | | | 15 | 64535700 | | | C | | 6.30E-06 | | 1.68E-03 | | | 0.15 | | 0.05 | 4.40E-01 | | | 0.07 | | 0.09 | | 1.76E-05 | 0.24 | | 0.06 | |
| Thomsen et al. 2019 | | rs74998556 | | | 17 | 16839782 | | | T | | 9.00E-07 | | 1.05E-06 | | | 0.36 | | 0.07 | - | | | - | | - | | 9.44E-05 | 0.38 | | 0.10 | |
| Thomsen et al. 2017 | | rs3131740 | | | 1 | 58225485 | | | C | | 1.20E-04 | | 2.89E-02 | | | 0.10 | | 0.04 | 8.02E-01 | | | 0.02 | | 0.08 | | 3.45E-01 | 0.14 | | 0.15 | |
| Thomsen et al. 2017 | | rs3118053 | | | 1 | 58226522 | | | T | | 9.70E-04 | | 5.77E-02 | | | 0.08 | | 0.04 | 5.73E-01 | | | 0.04 | | 0.08 | | 5.06E-01 | 0.10 | | 0.16 | |
| Thomsen et al. 2017 | | rs6933936 | | | 6 | 164043825 | | | T | | 5.10E-06 | | 2.53E-03 | | | 0.15 | | 0.05 | 4.74E-01 | | | 0.06 | | 0.09 | | 1.50E-01 | 0.21 | | 0.15 | |
| Thomsen et al. 2017 | | rs10251201 | | | 7 | 7972285 | | | C | | 2.80E-05 | | 3.00E-03 | | | 0.13 | | 0.04 | 6.47E-01 | | | 0.04 | | 0.08 | | 4.38E-02 | 0.23 | | 0.11 | |
| Thomsen et al. 2017 | | rs28381958 | | | 7 | 87164836 | | | A | | 1.30E-04 | | 7.59E-02 | | | 0.13 | | 0.07 | - | | | - | | - | | 4.88E-01 | 0.14 | | 0.20 | |
| Thomsen et al. 2017 | | rs974120 | | | 8 | 2646618 | | | C | | 4.50E-04 | | 9.30E-02 | | | 0.12 | | 0.07 | 6.63E-01 | | | 0.05 | | 0.12 | | 4.69E-01 | 0.26 | | 0.36 | |
| Thomsen et al. 2017 | | rs10744861 | | | 12 | 116122376 | | | T | | 2.90E-04 | | 9.66E-01 | | | 0.00 | | 0.07 | 1.10E-01 | | | 0.19 | | 0.12 | | 6.90E-01 | 0.10 | | 0.26 | |
| Thomsen et al. 2017 | | rs9318227 | | | 13 | 74500970 | | | T | | 3.90E-03 | | 4.02E-02 | | | 0.13 | | 0.06 | 8.62E-01 | | | 0.02 | | 0.11 | | 2.79E-01 | 0.24 | | 0.22 | |
| Thomsen et al. 2017 | | rs16966921 | | | 18 | 33205897 | | | G | | 3.70E-03 | | 4.30E-03 | | | 0.25 | | 0.09 | 3.03E-01 | | | 0.15 | | 0.15 | | 2.82E-01 | 0.29 | | 0.27 | |
| Thomsen et al. 2017 | | rs10405859 | | | 19 | 45602781 | | | T | | 1.50E-04 | | 6.71E-03 | | | 0.12 | | 0.04 | 9.31E-01 | | | 0.01 | | 0.08 | | 9.98E-02 | 0.19 | | 0.12 | |
| Went et al.  2018 | | rs7577599 | | | 2 | 25613146 | | | T | | 3.70E-09 | | 7.39E-01 | | | 0.02 | | 0.06 | 3.03E-04 | | | 0.37 | | 0.10 | | 5.19E-03 | 0.19 | | 0.07 | |
| Went et al.  2018 | | rs4325816 | | | 2 | 174808899 | | | T | | 1.20E-16 | | 3.50E-01 | | | 0.05 | | 0.05 | 7.69E-02 | | | 0.16 | | 0.09 | | 2.12E-02 | 0.14 | | 0.06 | |
| Went et al.  2018 | | rs6599192 | | | 3 | 41992408 | | | G | | 5.90E-15 | | 3.03E-02 | | | 0.13 | | 0.06 | 1.31E-02 | | | 0.25 | | 0.10 | | 2.69E-04 | 0.32 | | 0.09 | |
| Went et al.  2018 | | rs10936600 | | | 3 | 169514585 | | | A | | 8.70E-18 | | 6.88E-02 | | | 0.09 | | 0.05 | 7.15E-01 | | | 0.03 | | 0.09 | | 5.06E-02 | 0.12 | | 0.06 | |
| Went et al.  2018 | | rs1423269 | | | 5 | 95255724 | | | A | | 1.60E-11 | | 3.70E-01 | | | 0.04 | | 0.05 | 5.39E-01 | | | 0.05 | | 0.09 | | 1.43E-01 | 0.09 | | 0.06 | |
| Went et al.  2018 | | rs6595443 | | | 5 | 122743325 | | | T | | 1.20E-08 | | 6.20E-01 | | | 0.02 | | 0.04 | 3.34E-01 | | | 0.08 | | 0.08 | | 9.33E-01 | 0.01 | | 0.07 | |
| Went et al.  2018 | | rs34229995 | | | 6 | 15244018 | | | G | | 3.00E-17 | | - | | | - | | - | - | | | - | | - | | - | - | | - | |
| Went et al.  2018 | | rs3132535 | | | 6 | 31116526 | | | A | | 1.30E-08 | | 6.21E-01 | | | 0.02 | | 0.05 | 8.98E-03 | | | 0.21 | | 0.08 | | 2.30E-02 | 0.13 | | 0.06 | |
| Went et al.  2018 | | rs9372120 | | | 6 | 106667535 | | | G | | 9.10E-15 | | 6.90E-01 | | | 0.02 | | 0.05 | 3.71E-03 | | | 0.27 | | 0.09 | | 1.49E-01 | 0.09 | | 0.06 | |
| Went et al.  2018 | | rs4487645 | | | 7 | 21938240 | | | C | | 9.20E-09 | | 2.21E-02 | | | 0.10 | | 0.05 | 8.33E-01 | | | 0.02 | | 0.08 | | 8.09E-03 | 0.15 | | 0.06 | |
| Went et al.  2018 | | rs17507636 | | | 7 | 106291118 | | | C | | 5.30E-25 | | 2.18E-01 | | | 0.06 | | 0.05 | 1.46E-01 | | | 0.12 | | 0.09 | | 6.25E-01 | 0.03 | | 0.06 | |
| Went et al.  2018 | | rs58618031 | | | 7 | 124583896 | | | T | | 2.70E-08 | | 5.76E-01 | | | 0.03 | | 0.05 | 5.42E-01 | | | 0.05 | | 0.09 | | 4.84E-01 | 0.07 | | 0.10 | |
| Went et al.  2018 | | rs7781265 | | | 7 | 150950940 | | | T | | 9.70E-09 | | 1.34E-01 | | | 0.10 | | 0.07 | 3.14E-01 | | | 0.12 | | 0.12 | | 1.21E-01 | 0.19 | | 0.12 | |
| Went et al.  2018 | | rs1948915 | | | 8 | 128222421 | | | C | | 4.20E-11 | | 8.37E-02 | | | 0.08 | | 0.05 | 1.52E-01 | | | 0.11 | | 0.08 | | 1.93E-03 | 0.17 | | 0.06 | |

**Figure S1A. QQ plot of Meta-Analysis: MGUS GWAS**

Quantile-quantile plot from meta-analysis illustrating greater number of observed p-values with small values in the meta-analysis compared to expected. Also, the QQ plot notes that there was little evidence for inflation across the range of p-values, requiring no correction for inflation.

***
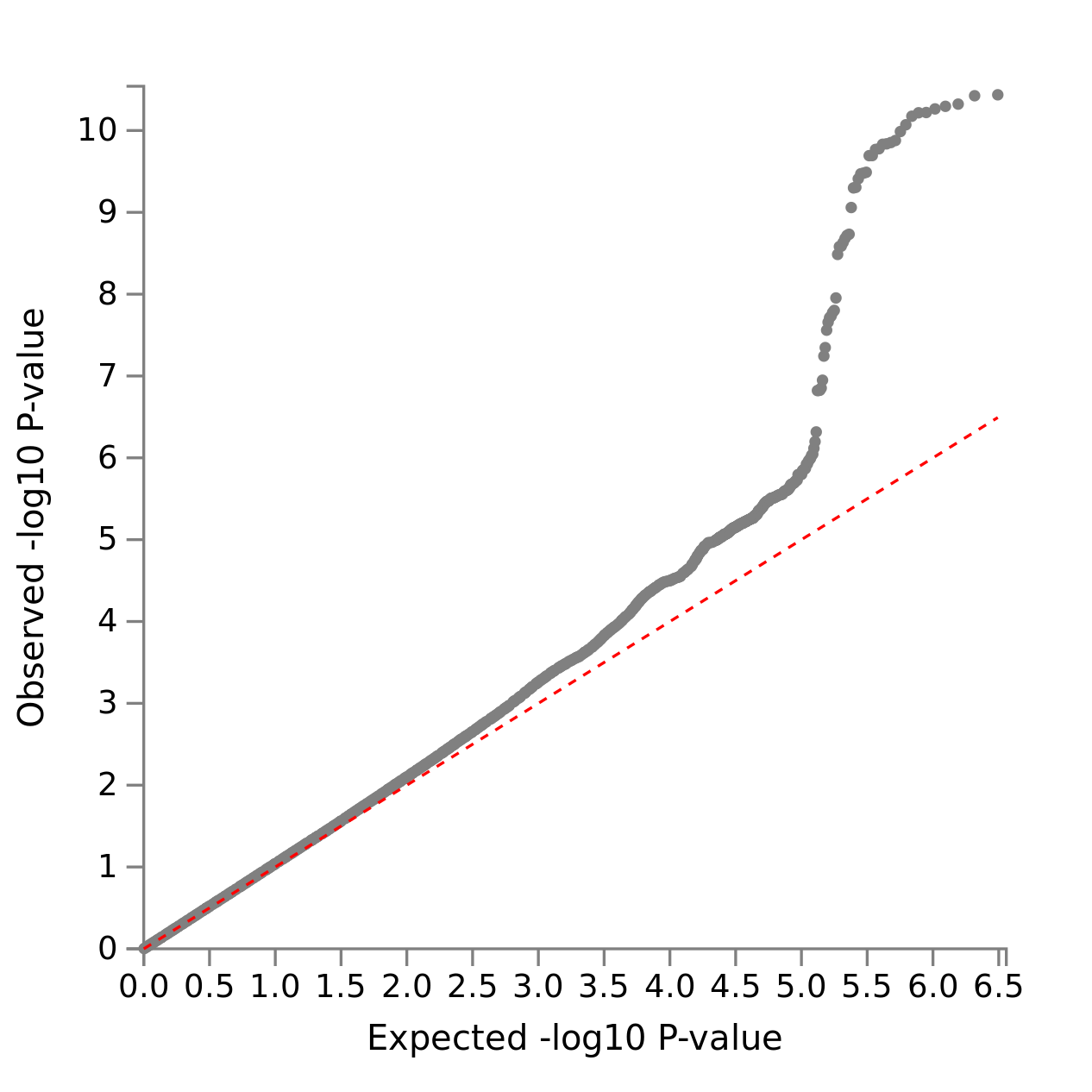
***

**Figure S1B.** **Regional association plots of genome-wide significant SNPs associated with MGUS risk.**

SNP associations (p-value) in the region around the genome-wide significant finding from the meta-analysis. The large red diamond indicates SNP rs195314, with the lowest p-value. Other diamonds note the level of linkage disequilibrium of SNPs in this region (orange in high linkage disequilibrium but blue is not).


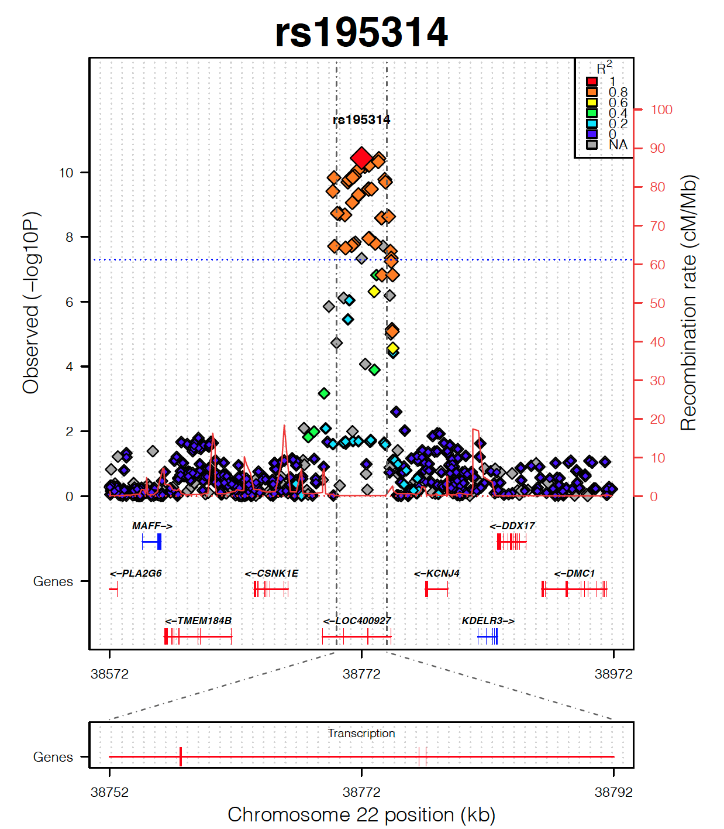

Supplement: Supplementary file 1 — Supplemental Material [file 41408_2022_658_MOESM1_ESM.docx]
